# Editorial: New Microbial Isolates From Hostile Environments: Perspectives for a Cleaner Future

**DOI:** 10.3389/fmicb.2021.740735

**Published:** 2022-01-04

**Authors:** Simona Di Gregorio, David B. Levin

**Affiliations:** ^1^Department of Biology, University of Pisa, Pisa, Italy; ^2^Department of Biosystem Engineering, University of Manitoba, Winnipeg, MB, Canada

**Keywords:** recalcitrant compounds, bioremediation, mycoremediation, genomics, fungal metabolism

In order to shift away from a fossil-based economy and mitigate climate change, many countries around the world support the transformation to a renewable, bio-based, and resource-efficient economy. Microorganisms are important actors in this direction. The number of described species of bacteria, and the amount of information about their metabolic capacities, is far greater than is available for fungi, and their biotechnological applications are extensively described in the scientific and technical literature. In contrast, Fungi are largely unexplored. Although the number of Fungi on the Earth is estimated to around six million species, only a very small percentage of these have been described scientifically, and even fewer are known in terms of genomes and metabolic potentials. A handful of species are understood well-enough to be used for applications in the biotechnology sector. Understanding fungal metabolic capacities and flexibilities is pivotal to achieve the bio-based and resource-efficient economy previously mentioned. Yeast have impacted society for millennia with the production of bread, beer, and wine. Fungal species capable of filamentous growth already offer additional beneficial properties such as the productions of a diverse array of metabolites, enzymes and materials.

We are currently in the era of the Anthropocene, an unprecidented period during which human activity is the dominant influence on climate and the environment. Evidence of environmental degradation due to the accumulation of waste materials from human activities is overwhelming. Much of this waste material consists of recalcitrant compounds that are resistant to natural biodegradation pathways. Pollution in soils and sediments is primarily due to total petroleum hydrocarbons (TPH) (Van Liedekerke et al., 2014), resulting from the chemical processing industry, leaks from underground hydrocarbon storage tanks, and accidental spills. The aromatic fraction of TPH is responsible for their recalcitrance to biodegradation (Booth et al., 2008). The selective pressure exerted by these contaminants leads to the adaptation of microorganisms capable of utilizing TPH components as carbon sources, resulting in the transformation, degradation, and eventually mineralization of these compounds (Margesin et al., 2003; Ron and Rosenberg, 2014; Lea-Smith et al., 2015). Microorganisms are able to both transform these toxic compounds as well as utilize them as energy sources, individually or under co-metabolic conditions, or they can decrease the toxicant bioavailability and stabilize them in the environment (Peng et al., 2008; Ghosal et al., 2016). The paper by Becarelli et al. exemplifies these processes. Becarelli et al. isolated a novel fungal species, in the genus *Cibora*, from hydrocarbon-polluted soil of an abandoned oil refinery in Italy. The strain was able to utilize diesel oil as a sole carbon source for growth. Laboratory-scale experiments were designed to evaluate the use of this fungal strain for treatment of the polluted soil. Functional metagenomic analyses revealed a number of generalist, saprophytic bacteria were essential for the onset of hydrocarbon degradation by specialist bacterial species involved in total petroleum hydrocarbon (TPH) depletion. The fungal metabolism accelerated the onset of specialist over generalist bacteria.

Monoaromatic hydrocarbons like xylenes are considered one of the most common hazardous sources of environmental contamination. Bacteria capable of degrading *m*-xylene or *p*-xylene under aerobic conditions are more common (Galli et al., 1992; Barbieri et al., 1993), while *ortho*-isomer degraders are less frequently reported due to its high recalcitrance. Among hydrocarbon-degrading bacteria, members of *Rhodococcus* genus represent a reservoir of intriguing genomic traits as well as functional diversity (Alvarez, 2019). Their importance is due to their metabolic and genetic flexibility and their tolerance to various stresses. Indeed, rhodococci are Gram-positive bacteria able to catabolize a remarkably wide range of organic and toxic compounds including *o*-xylene (Zampolli et al., 2019). Zampolli et al. employed RNA-seq analysis to elucidate the genetic determinants involved in the *o*-xylene degradation pathway in *R. opacus* R7. This approach identified several oxidative systems likely involved in *o*-xylene metabolism, and confirmed that *R. opacus* R7 possesses a redundancy of genes that contribute to *o*-xylene degradation. This work advances our understanding of *o*-xylene metabolism in bacteria belonging to *Rhodococcus* genus and provides a framework of useful enzymes (molecular tools) that can be fruitfully targeted for optimized *o*-xylene consumption.

Heavy metal pollution of the environment is a significant threat to the health of both people and animals. Copper (Cu) and Cobalt (Co) are among the most toxic heavy metals from mining and other industrial activities. In recent years several filamentous fungal strains have been isolated, identified and assessed for their heavy metal biosorption capacity for potential application in bioremediation of Cu and Co wastes (Dhankhar and Hooda, 2011; Akhtar and Mannan, 2020). Despite the growing interest in heavy metal removal by filamentous fungi, their exploitation faces numerous challenges such as finding suitable candidates for biosorption. Dusengemungu et al. review the current literature related to filamentous fungi with high metal uptake capacity, particularly for Cu and Co, as well as their future prospects.

Nitrate is a significant industrial pollutant in aqueous environment. High concentrations of nitrate in drinking water can cause life-threatening health problems, such as methanoglobanemia in infants and stomach cancer in adults). Therefore, wastewater treatment and aquaculture facilities are investing great efforts to develop highly efficient denitrification processes, and various technologies have been developed to improve the efficiency of nitrate removal (Bernhard, 2010; Zhang et al., 2015; Pan et al., 2017). Among these, biological removal of inorganic nitrogen compounds relies on the microorganisms populating the water treatment plants and the environmental conditions prevailing in the system. Furthermore, nitrifying and denitrifying bacteria generally require a relatively long hydraulic retention time (HRT) due to their slow growth rate. Therefore, stress events, low temperatures, continuous operation, and insufficient HRTs are likely to lead to the withdrawal and dilution of the nitrifying and denitrifying bacteria biomass from the treatment facility bioreactors (Joo et al., 2005; Chen et al., 2020). Shelly et al. isolated a novel species of bacteria, *Acinetobacter* EMY, and found that it effectively removed nitrate in cultures under anoxic conditions. Biofilms of *Acinetobacter* EMY attached to plastic biocarriers in batch and continuous moving bed bioreactors, demonstrated up to 1.75-times greater nitrate removal compared with bacteria in suspension. these results are among the highest values observed for nitrate removal, when compared with previous studies focused on the characterization of denitrifying bacteria, *Acinetobacter* EMY also consumed all of the organic carbon with the nitrate, without leaving any residuals of organic matter in the water, suggesting that this new isolate may provide a good solution for biological treatment of nitrate-polluted water.

The textile industry is globally distributed contributing with 7% of the total world exports, employing around 35 million workers, with most of them located in the developing countries with weak or no environmental protection regulations (Ali et al., 2018, 2020a,b). Although the economic importance of the textile industry is undeniable, it is responsible for an extensive list of environmental impacts. The most important are those resulting from the discharge of untreated effluents into the water bodies, which normally constitute 80% of the total emissions produced by this industry. The fashion industry, driven by a need to continuously provide new styles at very low prices, has led to a huge increases in the quantity of “disposable” clothes that are used for short periods of time and then discarded. Approximately 10,000 different dyes, with an estimated annual production of 280,000 tons, are commercially available worldwide; and azo dyes represent over 60% of the total dyes (Patel et al., 2017; Pattanaik et al., 2020). It is estimated that 20–50% of these dyes remain unfixed during the dyeing processes and ultimately end up in the dye effluents (Giovanella et al., 2020), leading to severe pollution of water supplies in the vicinity of dyeing industries (Neetha et al., 2019). In developing countries, these wastewaters are commonly used for the purpose of irrigation in agriculture, and pose a direct threat to human health.

It is significant, therefore, that Al-Tohamy et al. have isolated a new salt-tolerant yeast strain, *Sterigmatomyces halophilus* SSA-1575, for azo-dye decolorization and detoxification. This new strain was isolated from the gut of a wood-feeding termite, *Reticulitermes chinensis*. Under the optimized conditions, the *S. halophilus* SSA-1575 was able to completely decolorization the azo-dye Reactive Black 5 (at a concentration of 50 mg/L) within 24 h. Two enzymes, NADH-dichlorophenol indophenol (NADH-DCIP) reductase and lignin peroxidase (LiP) were found to be responsible for the decolourization activity, which was enhanced when the medium was supplemented with carbon and energy sources, such as glucose, ammonium sulfate, and yeast extract.

The rapid and humungous accumulation of synthetic plastics in the environment is yet another significant threat to life on our planet. Petroleum-derived (petro-)polymers such as polyethylene (PE), polyethylene terephthalate (PET), polyurethane (PU), polystyrene (PS), polypropylene (PP), and polyvinyl chloride (PVC) are extremely resistant to natural biodegradation pathways. Mohanan et al. have reviewed the growing literature on microbial and enzymatic degradation of synthetic polymers. Some microorganisms with the ability to degrade petro-polymers under *in vitro* conditions have been isolated and characterized, and in some cases, the enzymes expressed by these microbes have been cloned and sequenced. However, identifying enzymes that plastic-degrading microorganisms secrete, and understanding their mechanisms of synthetic polymer-degradation are just beginning to be systematically investigated. The review paper by Mohanan et al. provides a summary the advances made in the microbial degradation of synthetic plastics and overview the enzymes involved in biodegradation.

Development of a sustainable, circular bioeconomy is essential for the survival of our species and our civilization, and Fungi can play an important role in the development of biotechnologies dedicated to environment and sustainability. A greater understanding of fungal metabolism and the metabolic capacity encoded in fungal genomes will enable applications of these species to a wide-range of environmental remediation problems. This will require the application of “omic” sciences to exhaustively characterize organisms that may be used to drive and/or support the circular economy.

Our special topic in Frontiers in Microbiology, “New Microbial Isolates from Hostile Environments: Perspectives for a Cleaner Future,” describes the use of contaminated matrices as the source of microorganisms adapted to hostile environments. Extremotolerant organisms, which can tolerate extreme and/or toxic conditions are reasonably capable of adjusting, surviving, and/or thriving in contaminated, hostile habitats that are inhospitable, or even lethal, for life. The isolation, characterization, and description of microbes capable of surviving in hostile environments can provide metabolic pathways, enzymes, and biomolecules for diverse applications in biotechnology, including bioremediation and restoration of polluted environments.

## Author Contributions

SD prepared the first draft of the editorial. DL proof-read, edited, and revised the draft and serves as the corresponding author. Both authors contributed to the article and approved the submitted version.

## Conflict of Interest

The authors declare that the research was conducted in the absence of any commercial or financial relationships that could be construed as a potential conflict of interest.

## Publisher's Note

All claims expressed in this article are solely those of the authors and do not necessarily represent those of their affiliated organizations, or those of the publisher, the editors and the reviewers. Any product that may be evaluated in this article, or claim that may be made by its manufacturer, is not guaranteed or endorsed by the publisher.

## References

[B1] Akhtar N. Mannan M. A. (2020). Mycoremediation: expunging environmental pollutants. Biotechnol. Rep. 26:e00452. 10.1016/j.btre.2020.e00452 32617263 PMC7322808

[B2] Ali S. S. Al-Tohamy R. Sun J. Wu J. Huang M. (2018). The role of gut symbionts from termites: a unique hidden player from yeasts (Review). Acta Microbiol. Sin. 58, 1004–1015. 10.13343/j.cnki.wsxb.20170610

[B3] Ali S. S. Kornaros M. Manni A. Sun J. El-Shanshoury A.-E. R. Kenawy E.-R.. (2020a). Enhanced anaerobic digestion performance by two artificially constructed microbial consortia capable of woody biomass degradation and chlorophenols detoxification. J. Hazard. Mater. 389:122076. 10.1016/j.jhazmat.2020.122076 32004834

[B4] Ali S. S. Mustafa A. M. Kornaros M. manni A. Sun J. Khalil M. A. (2020b). Construction of novel microbial consortia CS-5 and BC-4 valued for the degradation of catalpa sawdust and chlorophenols simultaneously with enhancing methane production. Bioresour. Technol. 301:122720. 10.1016/j.biortech.2019.122720 31945685

[B5] Alvarez H. M.. (2019). Biology of Rhodococcus. Berlin: Springer. 10.1007/978-3-030-11461-9

[B6] Barbieri P. Palladino L. Di Gennaro P. Galli E. (1993). Alternative pathways for *o*-xylene or *m*-xylene and *p*-xylene degradation in *Pseudomonas stutzeri* strain. Biodegradation 4, 71–80. 10.1007/BF00702323

[B7] Bernhard A.. (2010). The nitrogen cycle: processes, players, and human impact. Nat. Educ. Knowl. 3:25. Available online at: https://www.nature.com/scitable/knowledge/library/the-nitrogen-cycle-processes-players-and-human-15644632/

[B8] Booth A. M. Scarlett A. G. Lewis C. A. Belt S. T. Rowland S. J. (2008). Unresolved complex mixtures (UCMs) of aromatic hydrocarbons: branched alkyl indanes and branched alkyl tetralins are present in UCMs and accumulated by and toxic to, the mussel *Mytilus edulis*. Environ. Sci. Technol. 42, 8122–8126. 10.1021/es801601x 19031912

[B9] Chen Z. Jiang Y. Chang Z. Wang J. Song X. Huang Z.. (2020). Denitrification characteristics and pathways of a facultative anaerobic denitrifying strain, *Pseudomonas denitrificans* G1. J. Biosci. Bioeng. 129, 715–722. 10.1016/j.jbiosc.2019.12.011 31974049

[B10] Dhankhar R. Hooda A. (2011). Fungal biosorption – an alternative to meet the challenges of heavy metal pollution in aqueous solutions. Environ. Technol. 32, 467–491. 10.1080/09593330.2011.572922 21877528

[B11] Galli E. Barbieri P. Bestetti G. (1992). Potential of *Pseudomonas* in the degradation of methylbenzenes, in Pseudomonas: Molecular Biology and Biotechnology, eds Galli E. Silver S. Witholt E. (Washington, DC: American Society for Microbiology).

[B12] Ghosal D. Ghosh S. Dutta T. K. Ahn Y. (2016). Current state of knowledge in microbial degradation of polycyclic aromatic hydrocarbons (PAHs): a review. Front. Microbiol. 7:1369. 10.3389/fmicb.2016.01369 27630626 PMC5006600

[B13] Giovanella P. GAL V. Otero I. V. R. Pellizzer E. de Jesus F. B.. (2020). Metal and organic pollutants bioremediation by extremophile microorganisms. J. Hazard. Mater. 382:121024. 10.1016/j.jhazmat.2019.121024 31541933

[B14] Joo H. -S. Hirai M. Shoda M. (2005). Characteristics of ammonium removal by heterotrophic nitrification-aerobic denitrification by *Alcaligenes faecalis* No. 4. J. Biosci. Bioeng. 100, 184–191. 10.1263/jbb.100.184 16198262

[B15] Lea-Smith D. J. Biller S. J. Davey M. P. Cotton C. A. Sepulveda B. M. P. Turchyn A. V.. (2015). Contribution of cyanobacterial alkane production to the ocean hydrocarbon cycle. Proc. Natl. Acad. Sci. U.S.A. 112, 13591–13596. 10.1073/pnas.1507274112 26438854 PMC4640736

[B16] Margesin R. Labbé D. Schinner F. Greer C. W. Whyte L. G. (2003). Characterization of hydrocarbon-degrading microbial populations in contaminated and pristine alpine soils. Appl. Environ. Microbiol. 69, 3085–3092. 10.1128/AEM.69.6.3085-3092.2003 12788702 PMC161509

[B17] Neetha J. N. Sandesh K. Kumar K. G. Chidananda B. Ujwal P. (2019). Optimization of direct blue-14 dye degradation by bacillus fermus (Kx898362) an alkaliphilic plant endophyte and assessment of degraded metabolite toxicity. J. Hazard. Mater. 364, 742–751. 10.1016/j.jhazmat.2018.10.074 30419543

[B18] Pan M. Huang X. Wu G. Hu Y. Yang Y. Zhan X. (2017). Performance of denitrifying phosphate removal *via* nitrite from slaughterhouse wastewater treatment at low temperature. Water 9:818. 10.3390/w9110818

[B19] Patel A. Sartaj K. Arora N. Pruthi V. Pruthi P. A. (2017). Biodegradation of phenol *via* meta cleavage pathway triggers *de novo* TAG biosynthesis pathway in oleaginous yeast. J. Hazard. Mater. 340, 47–56. 10.1016/j.jhazmat.2017.07.013 28711832

[B20] Pattanaik L. Duraivadivel P. Hariprasad P. Naik S. N. (2020). Utilization and re-use of solid and liquid waste generated from the natural indigo dye production process- a zero waste approach. Bioresour. Technol. 31:122721. 10.1016/j.biortech.2019.122721 31986372

[B21] Peng R. H. Xiong A. S. Xue Y. Fu X. Y. Gao F. Zhao W.. (2008). Microbial biodegradation of polyaromatic hydrocarbons. FEMS Microbiol. Rev. 32, 927–955. 10.1111/j.1574-6976.2008.00127.x 18662317

[B22] Ron E. Z. Rosenberg E. (2014). Enhanced bioremediation of oil spills in the sea. Curr. Opin. Biotechnol. 27, 191–194. 10.1016/j.copbio.2014.02.004 24657912

[B23] Van Liedekerke M. Prokop G. Rabl-Berger S. Kibblewhite M. Louwagie G. (2014). Progress in the Management of Contaminated Sites in Europe. Luxembourg: European Commission. 10.2788/4658

[B24] Zampolli J. Zeaiter Z. Di Canito A. Di Gennaro P. (2019). Genome analysis and -omics approaches provide new insights into the biodegradation potential of *Rhodococcus*. App. Microbiol. Biotechnol. 103, 1069–1080. 10.1007/s00253-018-9539-7 30554387

[B25] Zhang S. Sha C. Jiang W. Li W. Zhang D. Li J.. (2015). Ammonium removal at low temperature by a newly isolated heterotrophic nitrifying and aerobic denitrifying bacterium *Pseudomonas fluorescens* wsw-1001. Environ. Technol. 36, 2488–2494. 10.1080/09593330.2015.1035759 25827750

